# Short‐term resilience, long‐term costs: Reduced growth and increased erosion in the kelp *Ecklonia radiata* (phylum Ochrophyta) following repeated marine heatwaves

**DOI:** 10.1111/jpy.70076

**Published:** 2025-09-25

**Authors:** Olivia J. Wynn, Damon Britton, John Beardall, Cintia Iha, Allyson Nardelli, John A. Raven, Andrew Bridle, Catriona L. Hurd

**Affiliations:** ^1^ Institute for Marine and Antarctic Studies (IMAS) University of Tasmania (UTAS) Battery Point Tasmania Australia; ^2^ School of Biological Sciences Monash University Clayton Victoria Australia; ^3^ Australian National Algae Culture Collection (ANACC) Commonwealth Scientific and Industrial Research Organisation (CSIRO) Battery Point Tasmania Australia; ^4^ Division of Plant Science University of Dundee at the James Hutton Institute Dundee UK; ^5^ School of Biological Sciences University of Western Australia Crawley Western Australia Australia; ^6^ Climate Change Cluster University of Technology Sydney, Ultimo New South Wales Australia

**Keywords:** erosion, global ocean change, growth, kelp, marine heatwave, nitrate uptake, photosynthesis, physiology, pigment content, temperature

## Abstract

Marine heatwaves (MHWs) have increased in frequency by 34% since 1990 and are projected to rise further with global ocean change, posing significant risks to marine ecosystems. Kelps (order Laminariales) provide essential habitats and play key ecological roles, but they are increasingly threatened by MHWs. Tasmania, SE Australia, is a global warming hotspot, but the impacts of recurrent MHWs on the physiological performance of the ecologically dominant kelp *Ecklonia radiata* remain poorly understood. To address this, we investigated how the frequency of MHWs influenced the physiological and biochemical performance of *E. radiata*, both during and after MHWs, to evaluate immediate responses and recovery potential. In laboratory experiments, juvenile sporophytes were exposed to three experimental treatments: no‐MHW, a single 6‐day MHW, and double 6‐day MHWs followed by 7‐day recovery periods. *Ecklonia radiata* sporophytes were resilient to the single 6‐day MHW, but double MHWs negatively impacted recovery, with reduced growth rates and increased tissue erosion. Although photosynthetic rates remained unaffected, changes in pigment ratios and increased antioxidant activity indicated a mitigation of physiological stress. We propose that energy may be diverted from growth toward repair processes and the maintenance of essential functions. These findings suggest there was cumulative stress caused by repeated MHWs, leading to progressive physiological decline. More frequent MHW events may hinder *E. radiata*'s recovery capacity, with potential ecosystem implications, considering its key ecological role.

**Abbreviations**
DPPH: 2,2‐diphenyl‐1‐picryl‐hydrazyl
HSD: honestly significant difference
HW: heatwave
MHW: marine heatwave
SST: sea surface temperature
WW: wet weight

## INTRODUCTION

Due to anthropogenic climate change, the frequency of marine heatwaves (MHWs) has increased by 34% since 1990, increasing the number of global annual MHW days (Oliver, Donat, et al., 2018) and severely impacting marine ecosystems globally (Oliver et al., 2021; Smale et al., 2019). Marine heatwaves are defined as localized periods of extreme ocean warming that last for a duration of 5 or more consecutive days, with temperatures exceeding the 90th percentile of historical records (Hobday et al., 2016). In conjunction with ocean warming, the frequency of MHWs has been projected to further increase under anthropogenic climate change (Frölicher et al., 2018; International Panel on Climate Change, 2023; Smith et al., 2022). This could lead to enhanced negative impacts on ecosystems, particularly through disturbance, re‐structuring, and loss of important foundational species, such as corals, kelps, and seagrasses (Hughes et al., 2017; Oliver et al., 2021; Smale et al., 2019; Wernberg, 2021), potentially pushing ecosystems to the limits of their resilience.

Kelps (order Laminariales) are brown macroalgae that dominate temperate and subpolar rocky reef ecosystems (Krumhansl et al., 2016; Steneck et al., 2002; Wernberg et al., 2019). Kelp forests provide complex biogenic habitats, forming the base of diverse and productive ecosystems (Teagle et al., 2017). Marine heatwaves pose significant threats to kelp forests, potentially disrupting their structures and functions. Kelps are highly susceptible to these events, as illustrated by numerous global events of significant canopy loss, range contractions, and, in extreme cases, extinction (Rogers‐Bennett & Catton, 2019; Smale & Wernberg, 2013; Starko, Timmer, et al., 2024; Thomsen et al., 2019; Wernberg, 2021). These ecosystem shifts can lead to long‐term changes, including the dominance of turf algae, the creation of urchin barrens, or the establishment of invasive species (Filbee‐Dexter & Wernberg, 2018; Hobday et al., 2016; Ling, 2008; Oliver, Donat, et al., 2018; Serrao‐Neumann et al., 2016; Valentine & Johnson, 2004; Wernberg, Bennett, et al., 2016). Kelp mortality events potentially reduce genetic diversity but may have increased selection for future thermal performance within those populations (Coleman & Wernberg, 2020). However, these extreme events underscore the vulnerability of kelp forests to climate change‐induced MHWs.

Temperature is a key driver of kelp performance, influencing photosynthesis, growth, and biogeographic distribution (Davison, 1991; Eggert, 2012; Raven & Geider, 1988). Marine heatwaves are likely to push temperatures beyond kelp's thermal optima, leading to declines in performance through increased tissue erosion, reduced growth rates, and reduced photosynthesis (Andersen et al., 2013; Castro et al., 2024; Hawkins & Hartnoll, 1985; Leathers et al., 2023; Straub et al., 2019; Umanzor et al., 2021). Temperature effects occur at the cellular level, and increased temperatures alter protein function—affecting enzyme concentrations and enzymatic activity—and disrupt membrane structures, leading to alterations in ion transport and metabolic processes (Eggert, 2012). The severity and lasting impacts of MHWs will depend on the local environmental conditions, such as temperature and nutrient availability, an individual's critical thermal maximum (*CT*_max_), and MHW properties, such as frequency and severity (Eggert, 2012; Smale et al., 2019; Starko, van der Mheen, et al., 2024; Straub et al., 2019). The responses of kelp to warming and MHWs are highly dependent on the interactions of local environmental drivers and an individual's phenotypic plasticity (Fernández et al., 2020), determining responses of range expansion, acclimatization, adaptation, or mortality (Nauer et al., 2022). In the short term, kelps can employ a range of physiological acclimation mechanisms to mitigate the impacts of MHWs: (1) enhancing antioxidant production to manage oxidative stress (Fabbrizzi et al., 2023; Umanzor et al., 2021); (2) alternating metabolism (Nauer et al., 2022) for energy conservation or altering pigment concentrations, which maintain photosynthesis and provide antioxidant properties (Pérez‐Gálvez et al., 2020); and (3) adjusting physiological rate processes in response to increased temperatures, such as nutrient uptake rates, including increased active nitrate uptake (Roleda & Hurd, 2019). However, the increased energy reallocation to mitigation processes comes at an energetic cost to kelp performance (Davison, 1991; Eggert et al., 2012; Wahid et al., 2007).

Temperature increases, such as those caused by MHWs, impose significant energetic demands on kelps through mitigation of sublethal thermal stress (Davison, 1991). These energy trade‐offs, while allowing kelps to survive in the short term, have led to delayed negative impacts on their performance, such as increased bleaching, tissue erosion, and reductions in growth and photosynthesis during the recovery phase (Leathers et al., 2023; Nepper‐Davidsen, et al., 2019; Simonson et al., 2015). For example, *Laminaria ochroleuca*, a species observed across the northeast Atlantic, showed resilience during a MHW in the southwest United Kingdom (Bass et al., 2023) but experienced significant reductions in growth and photosynthesis, with increased bleaching following the MHW (Leathers et al., 2023), highlighting the importance of monitoring MHW recovery. These delayed impacts likely increase vulnerability to anthropogenic stressors, including recurrent MHWs. Successful recovery from an initial event is crucial for mitigating the cumulative damage from repeated heatwaves (HWs; Hatum et al., 2024). In other marine ecosystems, foundational species such as corals have demonstrated ecological memory, allowing them to better withstand recurrent thermal stress (Hughes et al., 2019). However, habitat‐forming species such as the octocoral *Eunicella singularis* have suffered cumulative damage from repeated thermal stress (Orenes‐Salazar et al., 2023). Although the effects of recurrent MHWs on kelp species remain understudied, the ability of kelp to withstand future events will depend on energy trade‐offs made to sustain stress mitigation mechanisms during recovery, further increasing their vulnerability to other environmental stressors over time. Although knowledge of these individual‐level physiological responses and recovery mechanisms is critical for predicting resilience, the responses also interact with population‐level processes, such as natural selection. Recurrent MHWs may act as selective pressures, favoring individuals with traits that enhance survival and recovery, thereby potentially shaping the genetic composition of kelp populations over time (Alsuwaiyan et al., 2021; Hu et al., 2020) ultimately whilst reducing genetic diversity (Gurgel et al., 2020). Understanding both individual and population‐level responses is essential for predicting their future resilience, given the critical structural role of kelp in coastal ecosystems (Straub et al., 2019).

*Ecklonia radiata*, a dominant brown kelp, is extensively distributed along approximately 8000 km of the Australian coastline, known as the Great Southern Reef. This range spans from the subtropical latitudes of Western Australia and Queensland to the temperate reefs of Tasmania (Wernberg et al., 2019). *Ecklonia radiata*'s broad geographic distribution has been attributed to its wide thermal tolerance, thriving in temperatures ranging from 7 to 26°C and surviving in vivo up to 28°C (Britton et al., 2024; Wernberg, De Bettignies, et al., 2016).

Tasmania has been identified as a global warming hotspot, warming at three to four times the global average due to the strengthening of the East Australian Current (EAC; Hobday & Pecl, 2014; Oliver, Lago, et al., 2018). Although observations of the impacts of MHWs on warm‐edge populations of kelp in Western Australia have been documented (Wernberg, Bennett, et al., 2016), there is limited knowledge regarding the responses of cool‐edge populations (Bennett et al., 2015). Models have predicted declines in cool‐edge populations of *Ecklonia radiata* in southeastern Victoria and Tasmania driven by ocean warming (Britton et al., 2024; Young et al., 2023); however, knowledge gaps remain in our understanding about the effect of the fluctuation of temperature through the increased frequency of MHW exposures on *E. radiata* physiology during and post‐HW (recovery phase). To address this, we investigated how HW frequency (no‐HW vs. single‐HW vs. double‐HW) affected the morphological, physiological, and biochemical responses of juvenile *E. radiata* sporophytes both during and after MHWs. Juvenile sporophytes were selected because early life‐history stages are often critical bottlenecks in the development of seaweed populations, making them particularly vulnerable to environmental stressors (Lotze et al., 2001). The HW temperature of 20°C was chosen based on prior studies on *E. radiata* juvenile sporophytes (Britton et al., 2024), which identified this temperature as ecologically relevant for eliciting physiological responses without exceeding lethal thresholds (Boyd et al., 2018) while still adhering to the definition of an MHW. Specifically, we examined whether repeated HW exposure led to cumulative stress or acclimation, and how these responses affected the potential for short‐term recovery. We hypothesized that: (1) growth rates would decline for double HW yet remain unchanged for single and no‐HW samples; (2) erosion would increase for the double HW treatment, particularly post‐HW; (3) photosynthesis and respiration rates would be similar for single and double‐HWs, regardless of sampling timepoint, as the thermal physiology of *E. radiata* is known (Britton et al., 2024); and (4) antioxidant metabolism would be upregulated following both a single and a double‐HW, but more so following the double HW.

## MATERIALS AND METHODS

### Sample collection and acclimatization

**FIGURE 1:**
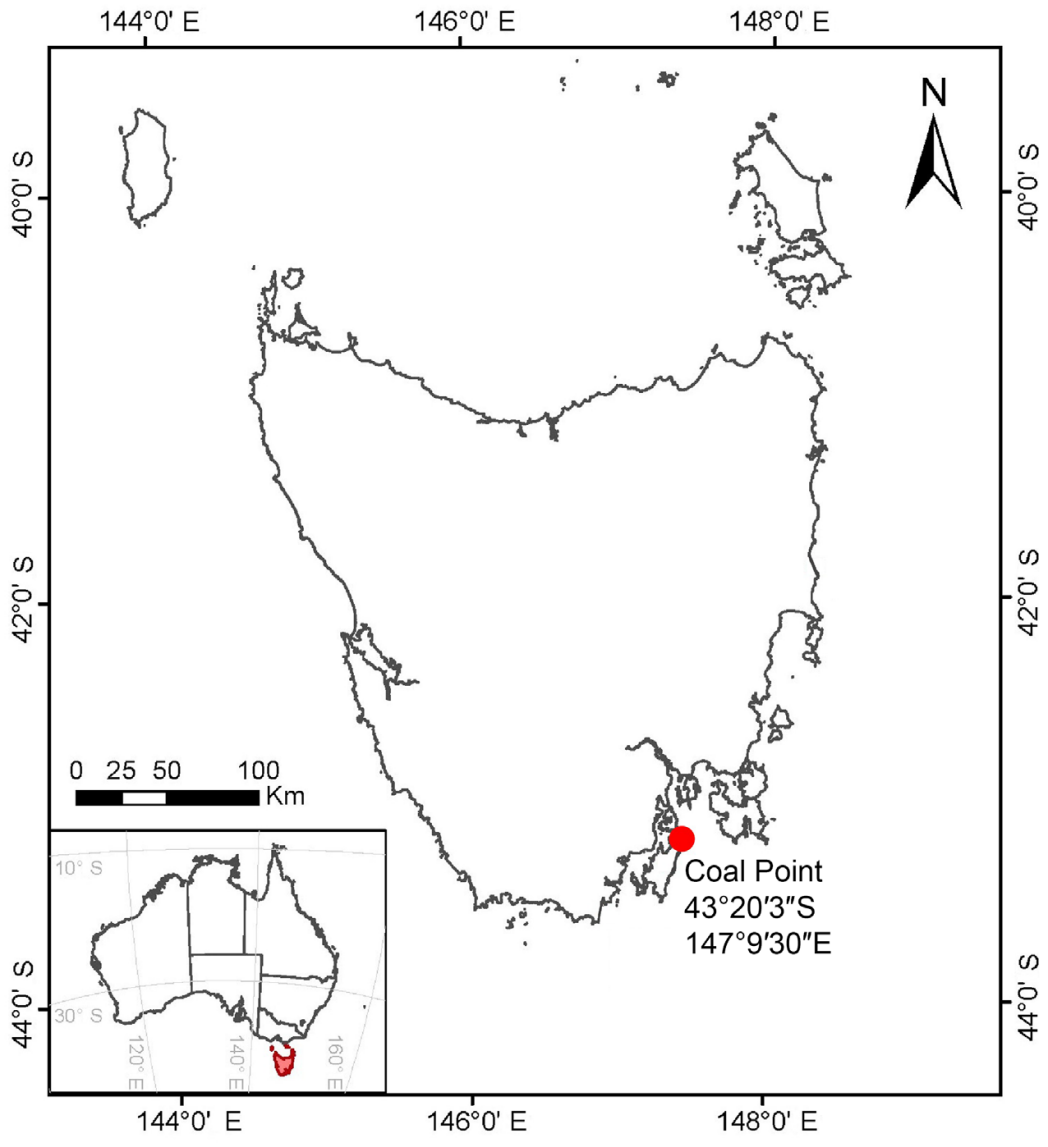
Geographic location of the study site at Coal Point, Bruny Island, in southeastern Tasmania, Australia (43°20′3″S, 147°19′30″E). The red dot represents the reef collection site.

On April 2020, 2023, approximately 70 juvenile sporophytes of *Ecklonia radiata* (~10–18 cm lengths) were collected from ~6 to 8 m depth at Coal Point, Tasmania (43°20′3″S, 147°19′30″E) by divers using SCUBA (Figure 1).

At the surface, sporophytes were placed in resealable bags containing seawater and transported to the laboratory, located 2 h away, in an insulated container. In the laboratory and to acclimate the seaweeds, the seaweeds were placed in a 30‐L container filled with filtered seawater (0.2‐μm pore size) in a temperature‐controlled room maintained at 14°C. The container was equipped with aeration for water motion and aquarium heaters (T100, Grant Instruments and Aqua One IPX8, Kongs Australia) set to 15°C, which was similar to the average sea surface temperature (SST) of 15.87°C from April 6, 2023, to April 20, 2023, at the collection site. The SST data were from a 0.02° daily L3‐gridded multisensor SST product provided by Australia's Integrated Marine Observing System (IMOS; https://imos.org.au/facility/satellite‐remote‐sensing/sea‐surface‐temperature‐products). LED lights (Zeus‐70, Ledzeal, Hong Kong) that replicated the color spectrum at approximately 8 m water depth were set to a 12:12 light:dark cycle with a max irradiance of 120 μmol photons · m^−2^ · s^−1^. Light was measured at the container surface using a LI‐COR Light Meter (LI‐193 Spherical Quantum Sensor, LI‐COR Biosciences, United States). Irradiance was increased linearly from 0 to 120 μmol photons · m^−2^ · s^−1^ between 06:00 and 11:00, was maintained at 120 μmol photons · m^−2^ · s^−1^ until 13:00, and irradiance was then decreased linearly back to 0 μmol photon · m^−2^ · s^−1^ by 18:00. This was controlled using the AquaZealer app (Shenzhen Topline Optoelectronic Co., Ltd., http://www.ledzeal.com/p47.html). The mean irradiance was 64 μmol photons · m^−2^ · s^−1^, and individuals were kept under these conditions for 48 h.

### Experimental culture conditions

**FIGURE 2:**
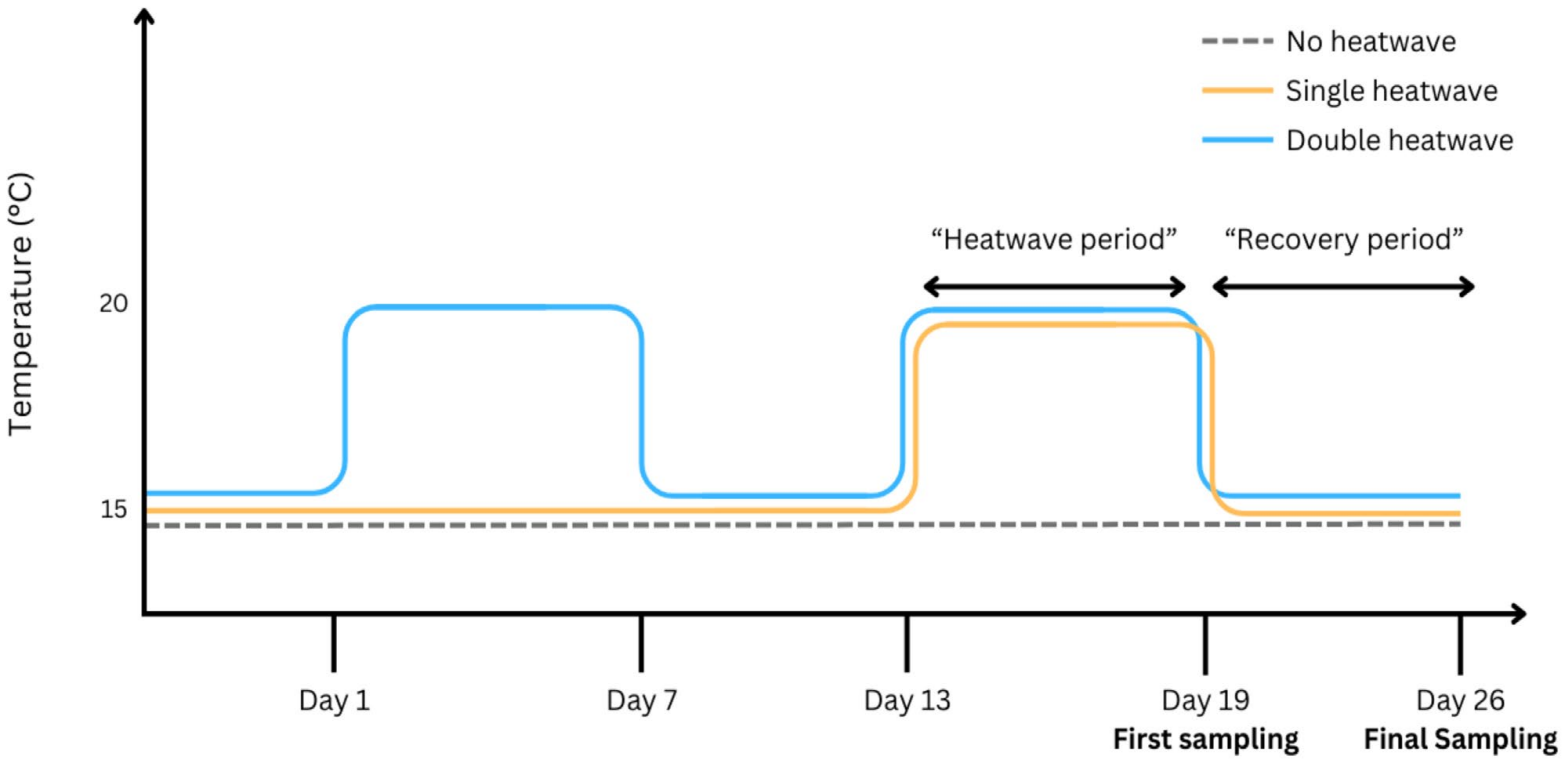
Experimental timeline of the sampling regime and the temperature scenarios in each heatwave treatment applied to juvenile *Ecklonia radiata* sporophytes. The first sampling was undertaken during the heatwave period on day 19, and the final sampling was undertaken during the recovery period on day 26. Lines representing the same temperature have been offset slightly to enhance visualization. Refer to the results for detailed temperature levels in each treatment. There were *n* = 9 replicates for each of six experimental treatment (*n* = 6): No‐HW during‐HW; single HW during‐HW; double HW during‐HW; no‐HW post‐HW; single HW post‐HW; double HW post‐HW.

Experiments were conducted in 2‐L culture chambers (see Britton et al., 2024). After 2 days of acclimatization to laboratory conditions, 54 individual juvenile *Ecklonia radiata* sporophytes were secured to a small rock (~40 mm ɸ) by their holdfast using a wide elastic band and then randomly assigned to 2‐L chambers containing 1.8 L of UV‐sterilized and filtered seawater (0.22‐μm pore size). Each chamber was randomly assigned to one of six treatments (see Figure 2). Experimental treatments consisted of three HW levels: no HW, a single HW, or a double HW. Heatwave treatments were crossed with two levels of sampling timepoint—during HW or post‐HW—with nine individuals for each combination of HW scenario and timepoint (*n* = 9). Treatments were applied by assigning chambers to temperature‐controlled water baths in a split‐plot design: three water baths for each HW treatment and timepoints split evenly between all nine baths (see Figure 2). The temperature of each water bath was initially set to the acclimatization temperature (15°C) with aquarium heaters (Jager 3612, Eheim, Germany) and maintained with temperature controllers (Inkbird ITC‐308S, China). Temperatures in each bath were recorded every 30 min using temperature loggers placed in the baths (HOBO Pendant MX temperature logger, Vietnam).

For the no‐HW treatment, temperatures were maintained at 15°C for the duration of the experiment. For the single‐HW treatment, temperature was maintained at 15°C for 13 days and then increased over 3 h to 20°C and left for 6 days to mimic a HW; following this, the temperature was reduced over 3 h to 15°C for a 7‐day “recovery period.” For the double‐HW treatment, on day 1 the temperature was increased over 3 h to 20°C and left for 6 days to mimic a HW; following this, temperature was reduced over 3 h to 15°C for a 7‐day recovery period. On day 13, the double HW temperature was increased again over 3 h to 20°C and left for another 6 days; following this, the temperature was reduced over 3 h to 15°C and left for 7 days. On the final day of the HW period, 27 individuals (*n* = 9 from each HW treatment) were removed for downstream analysis, representing the during‐HW timepoint. At the end of the recovery period (day 26), the final 27 individuals were removed (*n* = 9 from each HW treatment), representing the post‐HW timepoint. Figure 2 displays a timeline of treatment conditions and sampling times throughout the duration of the experiment. Biotic responses were analyzed to assess morphological, physiological, and biochemical performance. Net photosynthesis and respiration were measured for all individuals during the 24 h preceding their removal from culture (see below). Similarly, nitrate uptake rates were measured during the 48 h prior to removal (see below). The “HW period” and “recovery period” referred to in the rest of the manuscript are highlighted in Figure 2.

### Meristematic blade growth, change in blade length, and erosion

Meristematic growth, changes in blade length, and tissue erosion (tissue loss) were measured using the hole punch method (Mann & Kirkman, 1981). All parameters were assessed from photographs taken on day 1 of the experiment before placing the individuals in the culture chambers and again on the final sampling day, during the HW (day 19), and post HW (day 26). ImageJ software (Schneider et al., 2012) was used to analyze the photographs and calculate the following parameters:

$$\text{Meristematic growth}\;\left(G\right)={\text{Hole}}_{\text{Final}}-{\text{Hole}}_{\text{Initial}}$$

$$\text{Change in blade length}={\text{Length}}_{\text{Final}}-{\text{Length}}_{\text{Initial}}$$

$$\begin{array}{c} \text{Erosion}\;\left(\%\text{of initial length}\right)\\ =\left(\left(\left({\text{Length}}_{\text{Initial}}+G\right)–{\text{Length}}_{\text{Final}}\right)/\text{initial length}\right)\times 100 \end{array}$$

Change in wet weight (g WW · day^−1^) was calculated by:

$$\begin{array}{c} \text{Change in}\;\mathrm{wet}\;\text{weight}\;\left(g\;\mathrm{WW}\cdot {\mathrm{day}}^{-1}\right)\\ ={\text{Weight}}_{\text{Final}}-{\text{Weight}}_{\text{Initial}} \end{array}$$

Meristematic growth, change in blade length, and change in wet weight (WW) were standardized to days in the experiment.

We conducted linear regressions to assess the potential for bias in our growth parameter estimates, specifically to determine whether these parameters might have been under‐ or over‐estimated. This involved testing the relationship between the output parameter (e.g., change in blade length: mm · day^−1^) and the initial measurement (e.g., initial length: mm). By examining whether a significant relationship existed, we aimed to identify any systematic biases that could have influenced our results. The analysis revealed no significant correlations, suggesting that the initial measurements did not impact the accuracy of our growth estimates. Consequently, no adjustments to the data were necessary.

### Photosynthesis and respiration

Oxygen evolution (photosynthesis) in experimental light and oxygen consumption in the dark (respiration) were measured within sealed culture chambers, using nine replicates for each treatment, on days 18 and 19, respectively (final night and day of the HW period). The remaining nine replicates in each treatment were measured on days 25 and 26 (final night and day of the recovery period). To ensure the individuals were dark adapted, the lights were turned off 1 h before any respiration measurements were taken. Photosynthesis measurements were made between 10:30 and 19:03, and respiration measurements were made overnight, between 17:03 and 11:02. Measurements were made with a portable oxygen meter (Fibox 4, PreSens), coupled with a non‐invasive oxygen sensor in each culture chamber (Oxygen Sensor Spot SP‐PSt3‐NAU, PreSens). Net photosynthesis and respiration were expressed as μmol O_2_ · L^−1^ · cm^−2^ · h^−1^.

### Pigment content, antioxidant activity, and tissue C and N content

At the end of the experimental treatment, the thallus of each of the nine individual replicates was sampled and divided into four pieces for the following assays. One sample was preserved for gene expression work that is not included in this paper (Wynn et al., unpublished data). Tissue was immediately frozen at −20°C for pigment content and tissue C and N. Following freezing for C and N, tissue was freeze‐dried (FreezeZone 4.5, Labconco) and then remained at −20°C until analysis. Algal tissue for antioxidant activity assays was frozen at −80°C until analysis.

Photosynthetic pigments, fucoxanthin and chlorophyll content (*a* and *c*), were measured using preserved algal tissue (~0.15 g) and determined by the equations in Seely et al. (1972). Pigments were extracted as described in Stephens and Hepburn (2016) and Seely et al. (1972). Algal tissue was added to 4 mL of dimethyl sulfoxide (DMSO) in 15‐mL conical tubes (Eppendorf South Pacific Pty. Ltd.). The samples were vortexed and incubated in the dark for 30 min, and the absorbance of the supernatants was measured at 480, 582, 631, and 665 nm using a UV–VIS spectrophotometer (Halo RB‐10 UV–VIS ratio beam spectrophotometer, Dynamica Scientific Ltd.). Acetone (90% v/v, 6 mL) was added to the algal tissue, and the sample was vortexed then incubated in the dark for 30 min. The sample was centrifuged, and the supernatant absorbance was measured at 470, 581, 631, and 664 nm. Chlorophyll *a* and *c* and fucoxanthin were calculated from each extract (DMSO and Acetone), summed, and standardized to wet weight (g · g^−1^ WW). The ratios of chlorophyll *c* to chlorophyll *a* and fucoxanthin to chlorophyll *a* were calculated.

Antioxidants activity was assessed using the 2,2‐diphenyl‐1‐picryl‐hydrazyl (DPPH) radical scavenging assay, following the method outlined by Pires et al. (2017). Preserved algal tissue (200 mg) was ground into a fine powder in liquid nitrogen, then transferred to 1.5‐mL Eppendorf tubes. The assays were conducted in final volumes of 1 mL with methanol, and the samples were left in the dark at room temperature for 3 h to complete the extraction. Absorbances were measured by UV–VIS spectrophotometer (Halo RB‐10 UV–VIS ratio beam spectrophotometer, Dynamica Scientific Ltd., λ = 517 nm), and the results were displayed in percentage antioxidant activity (% AOX activity; Pires et al., 2017).

Tissue carbon (C), nitrogen (N), and C:N ratios were measured from freeze‐dried tissue. The tissue was ground into a fine powder using a Planetary Ball Mill (BM40, 50 Hz). The samples were analyzed using a NA1500 elemental analyzer coupled to a Thermo Scientific Delta V Plus via a Conflo IV at the Central Science Laboratory, University of Tasmania. The carbon and nitrogen content in the tissue was presented as a percentage of the dry weight, and the C:N ratio was expressed based on atomic weights.

### Nutrient uptake (nitrate)

Nitrate uptake experiments were run over 48 h and commenced on day 16 (during‐HW period, *n* = 27) and day 23 (post‐HW period, *n* = 27). Water samples were taken for nutrient analysis from each chamber 30 min after water changes (nutrient additions, 5 μM). Water samples were taken through a syringe, filtered through a 0.7‐μm GF/F filter, and frozen at −20°C in 12‐mL polyethylene nutrient tubes. To determine uptake rates, final water samples were taken 48 h later, on day 18 (during‐HW period, *n* = 27) and day 25 (post‐HW period, *n* = 27). Nitrate concentrations were determined using a QuikChem® 8500 Nutrient Analyzer (LaChat Instruments, Australia), and nitrogen uptake rates were standardized to surface area.

### Statistical analysis

All analyses were conducted using R v. 4.4.1 (R Core Team, 2022). To test for differences in all univariate response variables, a split‐plot design was used to assess responses to HW treatments across different sampling timepoints. The model included the fixed factors heatwave (three levels: no HW, single HW, and double HW), sampling timepoint (two levels: during‐HW and post‐HW), and their interaction. A temperature‐controlled water bath was included as a random factor. Model fits were evaluated to ensure they satisfied the assumptions of equal variances and normality of residuals using residual versus fitted plots and normal Q–Q plots. No transformations were required. All models were fit as linear mixed models using the lmer function in the lme4 package (Bates et al., 2015). Degrees of freedom were obtained using the Kenward‐Roger approximation, and *p*‐values were obtained using the lmertest package (Halekoh & Højsgaard, 2014). Differences in the main effect of the HW (α = 0.05) were compared using pairwise comparisons of each level of HW in the package emmeans (Lenth, 2023). When significant interactions were detected at α = 0.05, pairwise comparisons were conducted using Tukey's honestly significant difference (HSD) test to compare treatments within each timepoint using the emmeans package in R (Lenth, 2023). All pairwise comparisons used the Kenward‐Roger approximation for estimating degrees of freedom.

## RESULTS

### Experimental culture conditions

Average temperatures (±*SD*) outside of the HW period (including pre‐HW and recovery phases) for each treatment were as follows: no‐HW, 15.35 ± 0.18°C; single‐HW, 15.27 ± 0.34°C; and double‐HW, 15.27 ± 0.13°C. During the HW, average temperatures were as follows: no‐HW, 15.34 ± 0.19°C; single‐HW, 20.02 ± 0.38°C; and double‐HW, 20.09 ± 0.48°C.

### Growth and erosion

**FIGURE 3:**
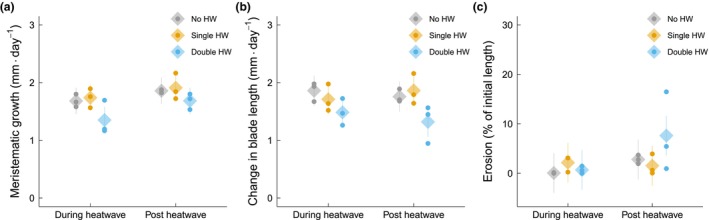
Growth and erosion of juvenile *Ecklonia radiata* cultured under different HW conditions: no HW (gray), single HW (orange), and double HW (blue). Samples were collected during (left) and post HW (right). Points represent raw data at the bath level, while diamonds depict mean estimates and lines show 95% confidence intervals, as predicted by the model. Response variables and significant differences are described for each panel. (a) Meristematic growth (mm · day^−1^); during HW < post HW (*p* = 0.015). (b) Change in blade length (mm · day^−1^); no HW > double HW (*p* = 0.039), single HW > double HW (*p* = 0.049). (c) Erosion (% of initial length); interaction significant (*p* = 0.022).

Meristematic growth was significantly higher in the recovery phase compared to during the HW (*p* = 0.015, linear mixed model, *F* = 6.40 on 1 and 42 degrees of freedom, 10% higher; Figure 3a). There was a marginally insignificant effect of HW treatment (*p* = 0.066, linear mixed model, *F* = 4.42 on 2 and 6 degrees of freedom), with a trend toward lower growth rates in the double‐HW treatment. Change in blade length was significantly affected by HW treatment (*p* = 0.029, linear mixed model, *F* = 6.75 on 2 and 6 degrees of freedom; Figure 3b), with the blade lengths of those subjected to the double‐HW treatment being significantly lower than those of both the no‐HW (20% lower, *p* = 0.039, Tukey's test, *t* = 3.27 on 6 degrees of freedom) and single‐HW (19% lower, *p* = 0.049, Tukey's test, *t* = 3.09 on 6 degrees of freedom) treatments. Erosion increased significantly in the recovery phase compared to the during‐HW phase (*p* = 0.007, Tukey's test, *t* = −2.85 on 42 degrees of freedom). These differences were driven by a significant interaction between HW treatment and time (*p* = 0.021, linear mixed model, *F* = 4.24 on 2 and 42 degrees of freedom; Figure 3c). Tukey's HSD post hoc tests indicated no differences between treatments within a sampling timepoint. Across timepoints, the erosion rate of *Ecklonia radiata* in the double‐HW treatment increased significantly in the recovery phase; it was 54% higher compared to the during‐HW phase (*p* = 0.003, Tukey's test, *t* = −3.22 on 42 degrees of freedom), while all other treatments showed no significant change across the timepoints. For change in wet weight (g WW · day^−1^), there was a significant interaction (*p* = 0.004, linear mixed model, *F* = 6.37 on 2 and 42 degrees of freedom; Figure S1), and Tukey's HSD post hoc tests indicated that during the recovery phase those exposed to two HWs had 28% lower WW than those from the no‐HW treatment (*p* = 0.025, Tukey's test, *t* = 2.94 on 15.8 degrees of freedom). Across timepoints, no‐HW samples increased WW in the recovery phase by 25% (*p* = 0.008, Tukey's test, *t* = −2.80 on 42 degrees of freedom); however, in the double‐HW treatment, WW declined by 21% (*p* = 0.036, Tukey's test, *t* = 2.17 on 42 degrees of freedom).

### Photosynthetic and respiration rates

**FIGURE 4:**
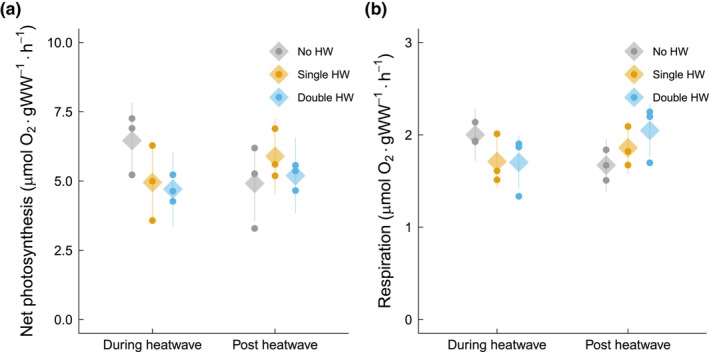
Photosynthesis and respiration of juvenile *Ecklonia radiata* cultured under different HW conditions: no HW (gray), single HW (orange), and double HW (blue). Samples were collected during (left) and post HW (right). Points represent the mean of the raw data at the bath level, while diamonds and lines depict the mean estimates and 95% the confidence intervals, respectively, as predicted by the model. Response variables and significant differences are described for each panel. (a) Net photosynthesis (μmol O_2_ · g^−1^ · h^−1^); significant interaction (*p* = 0.003). (b) Respiration (μmol O_2_ · g^−1^ · h^−1^).

Net photosynthetic rates of *Ecklonia radiata* in the no‐HW treatment declined significantly during the recovery phase compared to the during‐HW treatment samples (*p* < 0.005, Tukey's test, *t* = 2.97 on 42 degrees of freedom); although all other treatments showed no significant changes across the timepoints, there was a trend of the single HW increasing the net photosynthetic rate by 16% in the recovery phase (*p* = 0.08, Tukey's test, *t* = −1.82 on 42 degrees of freedom). These results indicate a significant interaction between HW treatment and sampling timepoint (*p* = 0.003, linear mixed model, *F* = 6.49 on 2 and 42 degrees of freedom; Figure 4a). Respiration rates remained stable across both HW treatments and timepoint (Figure 4b).

### Pigment content, antioxidant activity, and tissue C and N content

**FIGURE 5:**
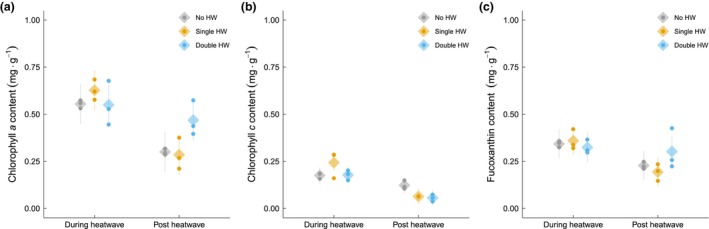
Pigment content of juvenile *Ecklonia radiata* cultured under different heatwave conditions: no HW (gray), single HW (orange), and double HW (blue). Samples were collected during (left) and post HW (right). Points represent the mean of the raw data at the bath level, while diamonds and lines depict mean estimates and 95% confidence intervals, respectively, as predicted by the model. Response variables and significant differences are described for each panel. (a) Chlorophyll *a* (mg · g^−1^); interaction significant (*p* = 0.007). (b) Chlorophyll *c* (mg · g^−1^); interaction significant (*p* = 0.006). (c) Fucoxanthin (mg · g^−1^); during HW > post HW (*p* < 0.001).

Chlorophyll *a* content was 39% lower in the recovery phase regardless of the HW scenario (*p* < 0.001, linear mixed model, *F* = 48.10 on 1 and 42 degrees of freedom; Figure 5a). A significant interaction was observed (*p* = 0.007, linear mixed model, *F* = 5.55 on 2 and 42 degrees of freedom), in which the no‐HW and single‐HW treatments showed reductions in the recovery phase compared to during the HW by 46% (*p* = 0.0001, Tukey's test, *t* = 4.51 on 42 degrees of freedom) and 55% (*p* < 0.0001, Tukey's test, *t* = 6.07 on 42 degrees of freedom), respectively. In the recovery phase, there was a trend indicating higher chlorophyll *a* content in the double‐HW treatment compared to both the no‐HW and single‐HW treatments by 36% (*p* = 0.08, Tukey's test, *t* = −2.41 on 12.7 degrees of freedom) and 39% (*p* = 0.052, Tukey's test, *t* = −2.63 on 12.7 degrees of freedom), respectively, although this was marginally insignificant. Chlorophyll *c* content was significantly lower in the recovery phase across all treatments (59% lower, *p* < 0.001, linear mixed model, *F* = 58.56 on 1 and 42 degrees of freedom; Figure 5b). A significant interaction (*p* = 0.006, linear mixed model, *F* = 5.72 on 2 and 42 degrees of freedom) revealed that single‐ and double‐HW treatments experienced reductions in the recovery phase compared to during the HW by 74% (*p* < 0.001, Tukey's test, *t* = 6.73 on 42 degrees of freedom) and 69% (*p* < 0.001, Tukey's test, *t* = 4.57 on 42 degrees of freedom), respectively. Fucoxanthin content was significantly lower in the recovery phase compared to during the HW (30% lower, *p* < 0.001, linear mixed model, *F* = 16.44 on 1 and 42 degrees of freedom; Figure 5c).

Chlorophyll *c*‐to‐chlorophyll *a* values in the recovery phase were 71% lower for the double‐HW treatment relative to the no‐HW treatment (*p* = 0.0003, Tukey's test, *t* = 4.77 on 21 degrees of freedom) and 56% lower for the double‐HW relative to the single‐HW treatments (*p* = 0.065, Tukey's test, *t* = 2.39 on 21 degrees of freedom). Chlorophyll *c*‐to‐chlorophyll *a* ratios declined over time in both the single‐HW treatment (*p* = 0.04, Tukey's test, *t* = 2.18 on 42 degrees of freedom) and the double‐HW treatment (*p* = 0.0003, Tukey's test, *t* = 3.91 on 42 degrees of freedom) by 33% and 67%, respectively. This resulted in a significant interaction between HW and timepoint (*p* = 0.003, linear mixed model, *F* = 6.93 on 2 and 42 degrees of freedom). The fucoxanthin‐to‐chlorophyll *a* ratio was significantly affected by timepoint, and ratios were 14% higher in the recovery phase relative to during the HW (*p* < 0.001, linear mixed model, *F* = 12.85 on 1 and 42 degrees of freedom).

**FIGURE 6:**
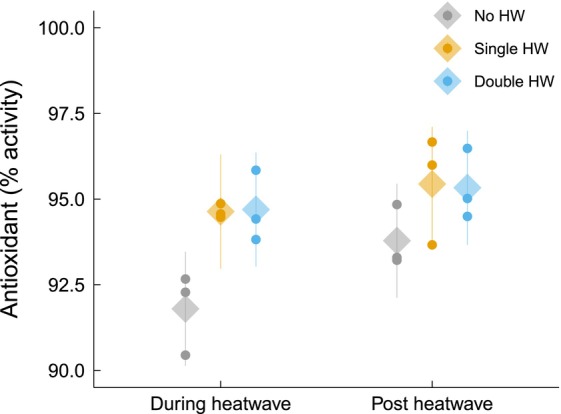
Antioxidant activity (%) of juvenile *Ecklonia radiata* cultured under different HW conditions: no HW (gray), single HW (orange), and double HW (blue). Samples were collected during (left) and post HW (right). Points represent the mean of the raw data at the bath level, while diamonds and lines depict mean estimates and 95% confidence intervals, respectively, as predicted by the model. Heatwave treatment significant (*p* = 0.0492).

**FIGURE 7:**
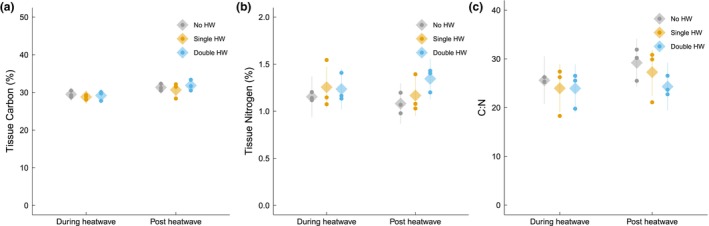
Tissue carbon (%), nitrogen (%) and C:N of juvenile *Ecklonia radiata* cultured under different HW conditions: no HW (gray), single HW (orange), and double HW (blue). Samples were collected during (left) and post HW (right). Points represent the mean of the raw data at the bath level, while diamonds and lines depict mean estimates and 95% confidence intervals, respectively, as predicted by the model. Tissue nitrogen and carbon are presented as % of algal weight. Response variables and significant differences are described for each panel. (a) Tissue nitrogen (%); no significant differences. (b) Tissue carbon (%); post HW > during HW (*p* < 0.001). (c) C:N; post HW > during HW (*p* = 0.006).

Antioxidant activity varied significantly among HW treatments (*p* = 0.049, linear mixed model, *F* = 5.19 on 2 and 6 degrees of freedom; Figure 6), with the activity associated with the no‐HW treatment being 2% lower than with the single‐HW and double‐HW treatments, regardless of timepoint. However, pairwise comparisons showed that these differences were marginally insignificant when comparing the no‐HW treatment to the single‐HW treatment (*p* = 0.069, Tukey's test, *t* = −2.81 on 6 degrees of freedom) and the double‐HW treatment (*p* = 0.072, Tukey's test, *t* = −2.77 on 6 degrees of freedom). Tissue N content did not differ significantly among treatments (Figure 7a). Tissue C content increased by 7% in the recovery phase compared to the during‐HW phase across all treatments, indicating a significant effect of time (*p* < 0.001, linear mixed model, *F* = 13.86 on 1 and 42 degrees of freedom; Figure 7b). Consequently, the tissue C:N increased by 9% in the recovery phase compared to the during‐HW phase (*p* = 0.006, Tukey's test, *t* = −2.92 on 42 degrees of freedom), supported by a significant effect of time (*p* = 0.006, linear mixed model, *F* = 8.54 on 1 and 42 degrees of freedom; Figure 7c).

### Nitrate uptake rate

**FIGURE 8:**
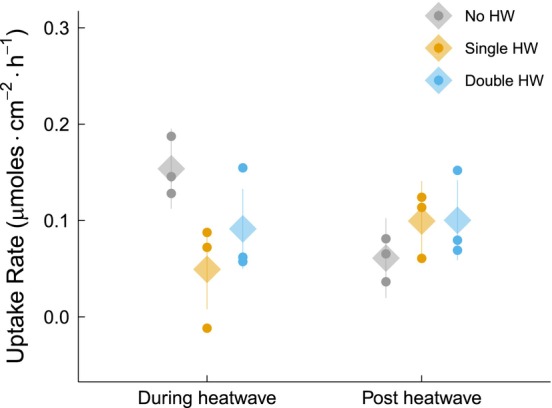
Nitrate uptake rate of juvenile *Ecklonia radiata* cultured under different HW conditions: no HW (gray), single HW (orange), and double HW (blue). Samples were collected during (left) and post HW (right). Points represent the mean of the raw data at the bath level, while diamonds and lines depict mean estimates and 95% confidence intervals, respectively, as predicted by the model. Nutrient uptake displayed as μmoles · cm^−2^ · h^−1^. Interaction significant (*p* = 0.002).

During the HW, nitrate uptake rates were significantly higher in the no‐HW treatment compared to the single‐HW treatment (68% higher, *p* = 0.0037, Tukey's test, *t* = 3.72 on 20 degrees of freedom) and showed a marginally insignificant trend of being higher than in the double‐HW treatment (41% higher, *p* = 0.091, Tukey's test, *t* = 2.22 on 20 degrees of freedom). In the recovery phase, nitrate uptake rates in the no‐HW treatment were lower than in both the single‐HW (39% lower) and double‐HW (40% lower) treatments, but these differences were not statistically significant. Across timepoints, the no‐HW treatment exhibited a significant 60% decrease in nitrate uptake rate in the recovery phase compared to the during‐HW phase (*p* = 0.0017, Tukey's test, *t* = 3.36 on 42 degrees of freedom). The single‐HW treatment showed a trend of increasing nitrate uptake rate by 40%, although this was marginally insignificant (*p* = 0.076, Tukey's test, *t* = −1.82 on 42 degrees of freedom), and the rates in the double‐HW treatment did not differ across timepoints. These differences in nitrogen uptake rates were driven by a significant interaction between HW treatment and sampling time (*p* = 0.002, linear mixed model, *F* = 7.10 on 2 and 42 degrees of freedom; Figure 8).

## DISCUSSION

Understanding the resilience of foundational kelp species to increasing HW events is critical for predicting the ecological impacts of climate change on marine ecosystems. We examined the effects of HW frequency—single or double events—on the growth, physiological, and biochemical performance of *Ecklonia radiata*, assessing both immediate responses during the HW(s) and the recovery potential after the events. By measuring a range of parameters under different HW scenarios on *E. radiata*, we assessed the potential for lasting impacts and whether HW frequency influenced resilience. Our results showed that juvenile *E. radiata* sporophytes were resilient to a single 6‐day HW, but a double HW had a negative impact, particularly during the recovery phase, with reduced growth rates and increased tissue erosion. Neither the single‐HW nor the double‐HW scenario affected photosynthetic rates; however, changes in pigment ratios and increased antioxidant activity were observed, indicating physiological stress mitigation (Collén & Davison, 1999; Davison et al., 1991; Rezayian et al., 2019). Notably, the single‐HW treatment reduced nitrate uptake rates during the HW but showed recovery post HW, whereas nitrate uptake rates remained unaffected by the double HW. These results highlight the potential for cumulative stress effects in *E. radiata* when exposed to repeated HWs that may lead to progressive physiological decline. The contrasting responses between single‐ and double‐HW treatments suggest that frequent HW events may compromise recovery of *E. radiata*. Coastal regions around southeastern Tasmania experience an average MHW duration of 6.87–7.05 days (Oliver, Donat, et al., 2018; Oliver, Lago, et al., 2018). It is important to recognize that longer‐lasting MHWs are likely to exacerbate the observed impacts, particularly under repeated exposures. Such prolonged events could result in more severe declines in growth and tissue integrity, further compromising recovery potential.

The growth and physiological responses of marine organisms to MHWs can lead to acute reversible or long‐lasting irreversible damage, both of which can be energetically costly to an individual (Oliver, Donat, et al., 2018; Oliver, Lago, et al., 2018; Smale et al., 2019; Wernberg et al., 2012). Our first hypothesis, that growth rates and change in blade length would decrease and blade erosion would increase in the double‐HW treatment compared to the single‐HW treatment, was mostly supported by our results. The growth rate remained unchanged after a single HW exposure, aligning with the expected thermal tolerance of cool‐edge populations of *Ecklonia radiata* (Britton et al., 2024). Although the optimum temperature for growth is 16°C, significant declines in growth rates have only occurred when temperatures have exceeded ~20°C (Britton et al., 2024). Blade length likely declined in the double‐HW treatment due to energy trade‐offs, in which energy may have been reallocated from growth to damage‐and‐repair mechanisms for maintaining essential processes, such as photosynthesis and cellular homeostasis (Davison, 1991; Eggert et al., 2012). In *Saccharina latissima* (Denamrk, Northeast Atlantic), simulated MHWs (+6°C) resulted in reduced growth rates that remained low in the recovery phase, resulting in a net loss of biomass (Nepper‐Davidsen et al., 2019). Similarly, although no changes in growth were observed in *E. radiata* during the double HW, declines became apparent post‐HW, suggesting short‐term thermotolerance mechanisms come at an energetic cost. This result paralleled results in *Laminaria ochroleuca* (southwest United Kingdom), in which significant post‐HW growth declines were observed despite no immediate effects during the HW (Leathers et al., 2023). Such delayed responses highlight the importance of post‐HW monitoring to understand the long‐term impacts of and recovery potential from thermal stress on kelp physiology.

The survival of kelp partly depends on the equilibrium between tissue growth and loss (Simonson et al., 2015). Distal erosion increased in *Ecklonia radiata* in the recovery phase after exposure to double HW, supporting our hypothesis. There has been supporting evidence of erosion under HW conditions for kelp in the laboratory (Bunting et al., 2024; Simonson et al., 2015; Straub et al., 2021) and in the field (Filbee‐Dexter et al., 2020). Endo et al. (2020) suggested that distal erosion under heat stress may be a mechanism that allows accumulation of N in the meristem, as observed in *Eisenia bicylis* juvenile sporophytes. Since kelp rely on N reserves that are stored during the winter months and utilized in summer (Chapman & Craigie, 1977; Chapman & Lindley, 1980), this process could be vital for maintaining N content. In our study, N content remained stable regardless of the treatment, suggesting that increased distal erosion may have facilitated the redistribution of N to replenish stores in the meristem. This result suggests that increased distal erosion may contribute to maintaining N reserves in vital areas such as the meristem. However, further research is needed to fully understand the implications of this process for kelp thermal physiology.

Photosynthesis and respiration remained stable regardless of treatment, whereas growth and erosion were negatively impacted following the double‐HW treatment. This indicated that although key metabolic processes were maintained, other physiological responses were more vulnerable to thermal stress. Previous studies on *Ecklonia radiata* have shown that photosynthesis remained stable until temperatures exceeded 22°C in cool‐edge populations, despite an optimum temperature of 18°C (Britton et al., 2024). In this study, HW temperatures were 20.02 ± 0.38°C and 20.09 ± 0.48°C for single‐ and double‐HW treatments, respectively, allowing *E. radiata* to maintain its photosynthetic capacity under double‐HW conditions. Similarly, *Macrocystis pyrifera* juvenile sporophytes (Baja California, Mexico) maintained net photosynthetic rates during HW conditions (22°C for 5 days), although respiration rates increased (Umanzor et al., 2021). Additionally, *Laminaria digitata* warm‐edge populations have exhibited increased photosynthetic rates; however, *L. hyperborea* (from mid‐range of the thermal limit) demonstrated stable rates under MHWs (Burdett et al., 2019). The maintenance of photosynthetic rates under MHWs may be due to a E. radiata’s ability to optimize electron transport through photosystem II or to activate thermal dissipation mechanisms, such as non‐photochemical quenching, to prevent photodamage (Deguette et al., 2022). Although these processes were not measured, it is likely that *E. radiata* possesses effective photoprotective mechanisms to cope with moderate thermal stress. This suggests that in the double‐HW exposure, *E. radiata* may have been reallocating energy produced through photosynthesis to stress‐mitigation and repair mechanisms rather than to growth. The stability of photosynthesis and respiration during the HW treatments highlights the resilience of the metabolic processes in *E. radiata*, although that resilience came at the cost of reduced growth and increased erosion. These results indicate that while photosynthesis was maintained under double‐HW conditions, metabolism may have been impacted, leading to trade‐offs in energy allocation. Energy has been diverted away from growth into stress‐mitigation mechanisms, including via antioxidant metabolism or by altering cell wall structure (not measured), as has been documented in *Phyllospora comosa* (Southeast Tasmania; Britton et al., 2020). However, further investigation including gene expression analyses is needed to elucidate these mechanisms.

One potential mechanism for coping with heat stress is the alteration in the proportions of photosynthetic pigments, which allows *Ecklonia radiata* to maintain photosynthetic efficiency despite environmental stressors. Specifically, we observed a 71% reduction in the ratio of chlorophyll *c* to chlorophyll *a* during the recovery phase after the double‐HW treatment compared to after the no‐HW treatment. This substantial decrease suggests a shift in pigment composition, likely as a response to thermal stress. A lower ratio of chlorophyll *c* to chlorophyll *a* indicates an increased reliance on chlorophyll *a* for light harvesting, potentially enhancing photosynthetic efficiency under stress by prioritizing the primary photosynthetic pigment. Similar adaptive responses have been documented in *Laminaria saccharina*: Elevated temperatures led to decreased ratio of chlorophyll *c* to chlorophyll *a*, reflecting a shift toward more efficient light harvesting by chlorophyll *a* (Davison et al., 1991). This strategy may enable *E. radiata* to optimize its photosynthetic apparatus under thermal stress conditions. In addition to pigment restructuring, our results showed a marginal increase in antioxidant activity in both the single‐ and double‐HW treatments compared to the no‐HW condition, partially supporting our hypothesis. The upregulation of antioxidant activity suggests that *E. radiata* engages its antioxidant defense mechanisms to scavenge excess ROS and prevent oxidative damage under heat stress (Collén & Davison, 1999; Rezayian et al., 2019). However, we had anticipated a more pronounced increase in antioxidant activity for the double‐HW treatment. One possible explanation is the limitation of the DPPH assay used, which may not fully have captured the antioxidant capacity due to its preference for non‐polar, organic‐soluble compounds (Floegel et al., 2011; Urrea‐Victoria et al., 2022). Consequently, our result might underestimate the total antioxidant response. Future research incorporating gene expression analysis and comprehensive profiling of antioxidant pathways would provide deeper insights into the mechanisms *E. radiata* employs to cope with heat stress.

## CONCLUSIONS

Marine heatwaves are becoming increasingly frequent, prolonged, and intense in Tasmania due to the intensification of the East Australian Current (Oliver, Lago, et al., 2018). Although *Ecklonia radiata* was able to maintain stable photosynthetic and respiratory rates during consecutive HW events, growth and tissue integrity, specifically erosion, were compromised during the recovery phase. This suggests that energy trade‐offs occurred, in which resources were redirected from growth to stress responses such as antioxidant production and repair. Over time, these trade‐offs may impact the long‐term fitness and resilience of the species. The delayed responses observed in *E. radiata* are consistent with patterns seen in other kelp species, such as *Laminaria ochroleuca* and *Saccharina latissima*, which have shown post‐HW declines in growth and photosynthesis (Leathers et al., 2023; Nepper‐Davidsen et al., 2019). This information highlights the need to monitor kelp performance beyond the immediate stress period to capture potential longer‐term impacts. The risk of cumulative damage from repeated MHWs further underscores the importance of understanding the thresholds at which resilience gives way to vulnerability (Hatum et al., 2024). Future molecular research will be key for revealing the underlying stress mitigation mechanisms, with the potential to explore genetic variability and epigenetic memory, as has been studied in other organisms (Lämke & Bäurle, 2017). Insights from such studies could inform conservation efforts aimed at safeguarding kelp forests in the face of intensifying climate change.

## AUTHOR CONTRIBUTIONS

**Olivia J. Wynn:** Conceptualization (equal); data curation (lead); formal analysis (equal); investigation (lead); methodology (equal); project administration (lead); writing – original draft (lead). **Damon Britton:** Conceptualization (equal); formal analysis (equal); methodology (equal); supervision (supporting); visualization (equal); writing – review and editing (equal). **John Beardall:** Funding acquisition (equal); resources (equal); writing – review and editing (equal). **Cintia Iha:** Supervision (supporting); writing – review and editing (equal). **Allyson Nardelli:** Methodology (supporting); writing – review and editing (equal). **John A. Raven:** Funding acquisition (equal); resources (equal). **Andrew Bridle:** Conceptualization (equal); funding acquisition (equal); resources (equal). **Catriona L. Hurd:** Conceptualization (equal); funding acquisition (equal); methodology (equal); project administration (equal); resources (equal); supervision (lead); writing – review and editing (equal).

## FUNDING INFORMATION

The research was funded by an Australian Research Council Discovery Project DP200101467 attributed to Catriona L. Hurd, John Beardall, Andrew Bridle, and John A. Raven.

## Supporting information


**Figure S1.** Change in wet weight (mg · day^−1^) of juvenile *Ecklonia radiata* cultured under different HW conditions: no HW (gray), single HW (orange), and double HW (blue). Samples were collected during (left) and post HW (right). Points represent the mean of the raw data at the bath level, while diamonds and lines depict mean estimates and 95% confidence intervals, respectively, as predicted by the model. Heatwave treatment significant (*p* = 0.0492).
