# Supplementary figures and images for: Short‐term resilience, long‐term costs: Reduced growth and increased erosion in the kelp Ecklonia radiata (phylum Ochrophyta) following repeated marine heatwaves
Source: J Phycol. 2025 Sep 25;61(5):1355–70. doi: 10.1111/jpy.70076 (PMC12547646; doi:10.1111/jpy.70076)

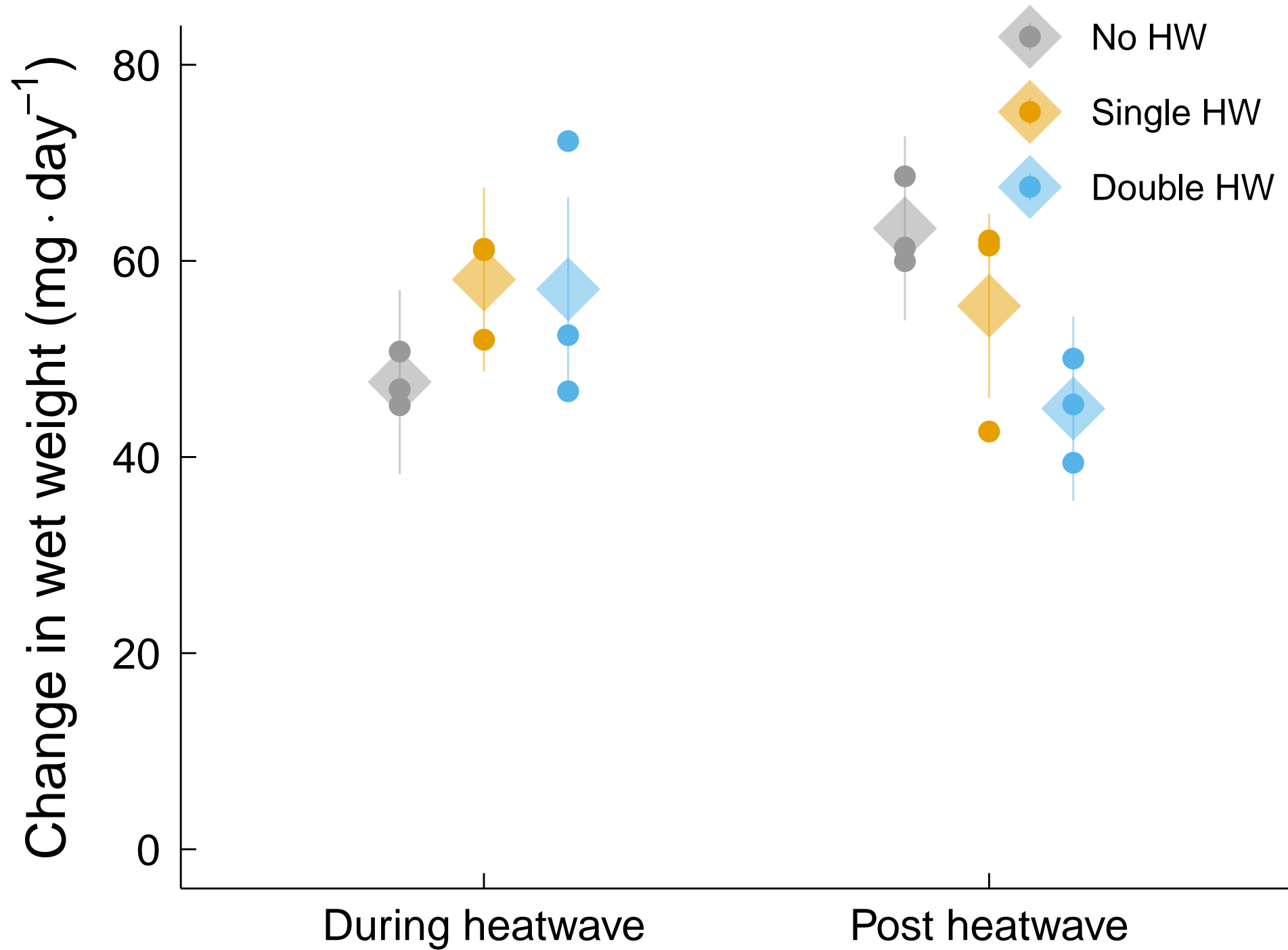

Supplement: Supplementary file 1 — Figure S1. Change in wet weight (mg · day−1) of juvenile Ecklonia radiata cultured under different HW conditions: no HW (gray), single HW (orange), and double HW (blue). Samples were collected during (left) and post HW (right). Points represent the mean of the raw data at the bath level, while diamonds and lines depict mean estimates and 95% confidence intervals, respectively, as predicted by the model. Heatwave treatment significant (p = 0.0492). [file JPY-61-1355-s001.pdf]
